# Comparative Study of the Efficiency of Different Noble Metals Supported on Hydroxyapatite in the Catalytic Lean Methane Oxidation under Realistic Conditions

**DOI:** 10.3390/ma14133612

**Published:** 2021-06-28

**Authors:** Zouhair Boukha, Beatriz de Rivas, Juan R. González-Velasco, José I. Gutiérrez-Ortiz, Rubén López-Fonseca

**Affiliations:** Chemical Technologies for Environmental Sustainability Group, Chemical Engineering Department, Faculty of Science and Technology, University of The Basque Country UPV/EHU, 48940 Leioa, Bizkaia, Spain; beatriz.derivas@ehu.eus (B.d.R.); juanra.gonzalezvelasco@ehu.eus (J.R.G.-V.); joseignacio.gutierrez@ehu.eus (J.I.G.-O.); ruben.lopez@ehu.eus (R.L.-F.)

**Keywords:** noble metals, hydroxyapatite, lean methane oxidation, OSC, water and CO_2_ effects

## Abstract

The combustion of lean methane was studied over palladium, rhodium, platinum, and ruthenium catalysts supported on hydroxyapatite (HAP). The samples were prepared by wetness impregnation and thoroughly characterized by BET, XRD, UV-Vis-NIR spectroscopy, H_2_-TPR, OSC, CO chemisorption, and TEM techniques. It was found that the Pd/HAP and Rh/HAP catalysts exhibited a higher activity compared with Pt/HAP and Ru/HAP samples. Thus, the degree of oxidation of the supported metal under the reaction mixture notably influenced its catalytic performance. Although Pd and Rh catalysts could be easily re-oxidized, the re-oxidation of Pt and Ru samples appeared to be a slow process, resulting in small amounts of metal oxide active sites. Feeding water and CO_2_ was found to have a negative effect, which was more pronounced in the presence of water, on the activity of Pd and Rh catalysts. However, the inhibiting effect of CO_2_ and H_2_O decreased by increasing the reaction temperature.

## 1. Introduction

The implementation of strict environmental regulations imposes the use of more efficient and cleaner fuels. In this sense, the utilization of lean burn natural gas (NG) engines is one of the most attractive strategies compared to those based on gasoline or diesel [1,2,3,4,5,6]. In addition to its high calorific value, the combustion of this fuel results in advantageous CO_2_ emissions and emits lower levels of toxic CO and NO_x_. However, the release of unburnt methane presents a significant problem that contributes to the greenhouse effect. Though it is less abundant in the atmosphere, methane is significantly more active in trapping radiation than CO_2_.

As a result, it is essential that active and durable catalysts are developed for CH_4_ emission abatement. It is known that noble metals exhibit a high activity and good resistance to coke formation [1,4,5,6,7]. Among the noble metals, Pd-based catalysts supported on alumina are clearly the most investigated systems. Nevertheless, few catalysts have demonstrated a reasonable activity and long-term stability under realistic reaction mixtures, in the presence of large amounts of H_2_O (10–15%) and CO_2_ (10–15%), and trace levels of sulfur compounds (SO_2_ or H_2_S) present in the exhaust gas of NG engines [4]. To address these major disadvantages, current research is focused on the use of alternative supports that present suitable interactions with the active phase [1,2,6,7,8,9]. Similarly, various promoters and bimetallic formulations have been examined to improve the activity and the resistance of the catalysts [10,11,12,13].

Numerous reports have claimed that the oxidation state of the catalyst plays a key role in controlling its performance for methane oxidation. There is broad consensus that the active sites are generally composed of the metal oxide [1,4,7,13]. However, the occurrence of oxygen vacant sites in the proximity of the active phase or at the metal–support interface also seems to be extremely important. This confers to the catalyst a significant ability to undergo redox process and oxygen mobility, in accordance with the Mars–Van Krevelen mechanism. Cullis et al. [13] demonstrated that the nature of the used support influenced the ability of noble metals to adsorb oxygen, and there was a correlation between the oxygen adsorption capacity of the supported precious metal and its catalytic activity for methane oxidation.

Due to their distinct properties, HAP materials have attracted considerable attention as catalyst supports for numerous applications [14,15,16,17,18,19,20,21,22,23,24]. Previous reports noted that the advantage of the use of HAP as a support lies in its capacity to retain its structural properties when it undergoes significant changes in composition [7,24]. The promising results arising from the use of HAP were found to be connected to its high thermal stability, acid-base properties, and the fact that it could provide beneficial synergistic effects with a variety of metallic active phases [14,15,16,17,18,19,20,21,22,23,24].

In the present study, we examined the suitability of HAP as a support for a series of noble metal catalysts (Pd, Rh, Pt and Ru) for the methane oxidation reaction. The catalysts were extensively characterized by BET, XRD, TEM, H_2_-TPR, UV-Vis-NIR spectroscopy, CO chemisorption, and OSC techniques to correlate their activity with the structural features. To the best of our knowledge, no similar comparative study has been conducted on the activity of the series of noble metal catalysts supported on HAP.

## 2. Materials and Methods

### 2.1. Preparation of the Catalysts

The HAP support was synthesized using the co-precipitation method, adding drop wise a calcium nitrate solution to a solution of (NH_4_)_2_HPO_4_ in a basic medium (pH = 10). Under stirring, the mixture was heated to 80 °C and then maintained for 16 h to accelerate its crystallization process. After filtration, the recovered solid was washed with distilled water, dried overnight at 120 °C, and then calcined in static air at 500 °C for 4 h.

The noble metal catalysts (Pd/HAP, Rh/HAP, Pt/HAP, and Ru/HAP), with a metal loading of 0.5 wt.%, were prepared by incipient wetness impregnation of the support with aqueous solutions of the respective precursor salts; namely Pd(NH_3_)_4_Cl_2_·H_2_O, RhCl_3_∙3H_2_O, [Pt(NH_3_)_4_](NO_3_)_2_, and Ru(NO)(NO_3_)_3_, respectively. The impregnated samples were dried overnight at 120 °C and then calcined at 500 °C for 4 h.

### 2.2. Characterization Techniques

The textural properties of the prepared catalysts were investigated by N_2_ physisorption experiments at −196 °C on a Micromeritics (TRISTAR II 3020) apparatus (Micromiritics Instrument Corp, Norcross, GA, USA). Prior to analysis, the samples were purged with N_2_ flow at 300 °C for 8 h. The structural properties were investigated by XRD. The analyses were performed on a X’PERT-MPD X-ray instrument (Malvern Panalytical Ltd., Royston, UK). The coordination and the oxidation state of the metallic species were studied by diffuse reflectance UV-Vis-NIR spectroscopy with a Cary 5000 apparatus (Agilent Technologies, Santa Clara, CA, USA).

Temperature-programmed reduction with hydrogen (H_2_-TPR) experiments were carried out on a Micromeritics AutoChem 2920 apparatus (Micromiritics Instrument Corp. Norcross, GA, USA). The samples were pre-treated in a flow of 5% O_2_/He at 500 °C for 30 min and then cooled to −30 °C in He. Finally, they were reduced in 5% H_2_/Ar gas flow (50 cm^3^ min^−1^) by increasing the temperature from −30 to 750 °C with a ramp of 10 °C min^−1^.

The size and dispersion of the metallic particles for the reduced samples were investigated by transmission electron microscopy (TEM). The observations were performed on a TECNAI G2 20 TWIN microscope (FEI Company, Hillsboro, OR, USA) operated at 200 kV and equipped with a LaB6 filament. The metallic dispersion was also estimated by CO chemisorption at 40 °C using the same instrument used for H_2_-TPR studies. The samples were pre-reduced by flowing 5% H_2_/Ar (50 cm^3^ min^−1^) at 400 °C for 1 h. Then, they were cooled under He flow to 40 °C and submitted to a series of CO pulses (using 5% CO/He and a loop volume of 0.5 cm^3^) until their saturation. The metallic dispersion, defined as the exposed active metal fraction, was determined on the assumption of a unity adsorption stoichiometry.

The oxygen storage capacity (OSC) for the synthesized samples was studied by oxygen volumetric chemisorption in the temperature range of 350–500 °C on a Micromeritics AutoChem 2920 instrument (Micromiritics Instrument Corp. Norcross, GA, USA). The samples (100 mg) were pre-reduced with a flow of 5% H_2_/Ar (50 cm^3^ min^−1^) at 500 °C for 1 h and, then, evacuated in He for 1 h. After their cooling to the analysis temperature, they were submitted to twenty O_2_ pulses (5% O_2_/He, loop volume: 0.5 cm^3^), injected in He carrier (50 cm^3^ min^−1^).

### 2.3. Catalytic Performance Testing

The catalytic runs were carried out in a fixed-bed reactor operating at atmospheric pressure. The catalysts (200 mg, 160–250 μm) diluted with quartz particles were pre-treated under a 5% O_2_/He flow (100 cm^3^ min^−1^) at 500 °C for 1 h. The standard reaction mixture was composed of 1%CH_4_ and 20%O_2_ balanced with He (F_tot_ = 100 cm^3^ min^−1^) corresponding to a weight hourly space velocity (WHSV) of 300 cm^3^ CH_4_ h^−1^ g^−1^. The reaction temperature was increased from 200 to 500 °C with a ramp of 1 °C min^−1^. The thermocouple was positioned at the inlet of the catalyst bed. Additional experiments were performed to study the influence of the addition of water (10%) and CO_2_ (10%). The water vapor was fed using a GILSON 307 pump (Gilson Inc., Middleton, OR, USA). The analysis system consisted of a gas chromatograph (Agilent Technologies 490 Micro GC) (Agilent Technologies, Santa Clara, CA, USA) equipped with a TCD detector.

## 3. Results

### 3.1. Characterization of the Catalysts

The N_2_ adsorption/desorption isotherms corresponding to the bare support and the four noble metal-modified samples, respectively, are presented in Supplementary Materials Figure S1. All samples exhibit similar shapes of both the isotherms and hysteresis loops. The adsorption branches are analogous to those of type II whereas the desorption isotherms present a hysteresis loop of H3, typically found over aggregates presenting slit-shaped pores. Data from Table 1 reveal that the modification of the HAP support with the noble metals does not provoke significant changes in its textural properties. The measured specific surface areas were found to range between 48 and 53 m^2^ g^−1^ (55 m^2^ g^−1^ for the bare support).

**Figure 1:**
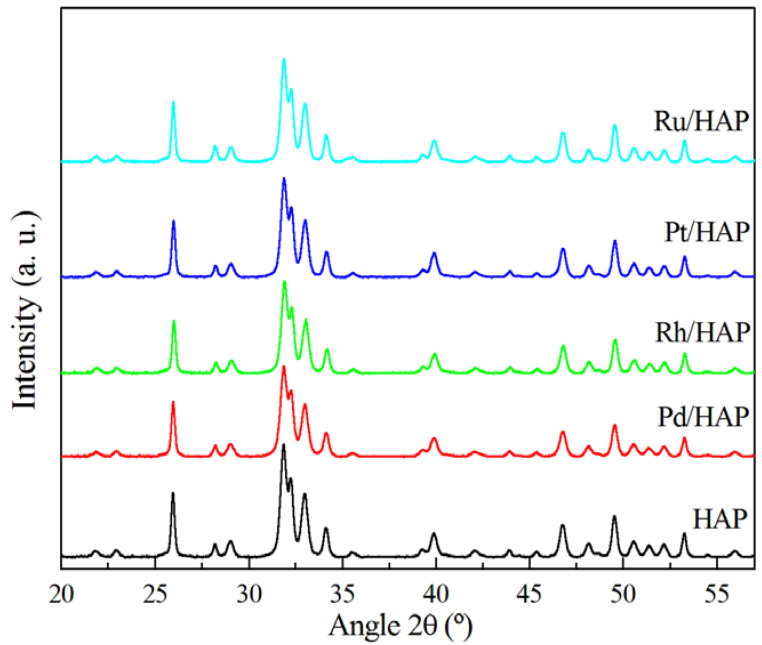
XRD patterns of the noble metal catalysts.

The XRD pattern of the HAP bare support (Figure 1) shows that the positions of all diffraction peaks are identical to those expected for the hydroxyapatite structure (JCPDS 01-082-2956), which crystallizes in an hexagonal system and belongs to the P6_3_/m space group. The addition of noble metals (Pd, Rh, Pt, and Ru) to the HAP and the successive thermal treatment at 500 °C does not significantly affect the structure of the support and its lattice parameters (Supplementary Materials Table S1). Moreover, no additional diffraction peaks due to the deposited metals are observed, which can be explained by their small amounts (0.5 wt.%) and/or their high dispersion.

**Figure 2:**
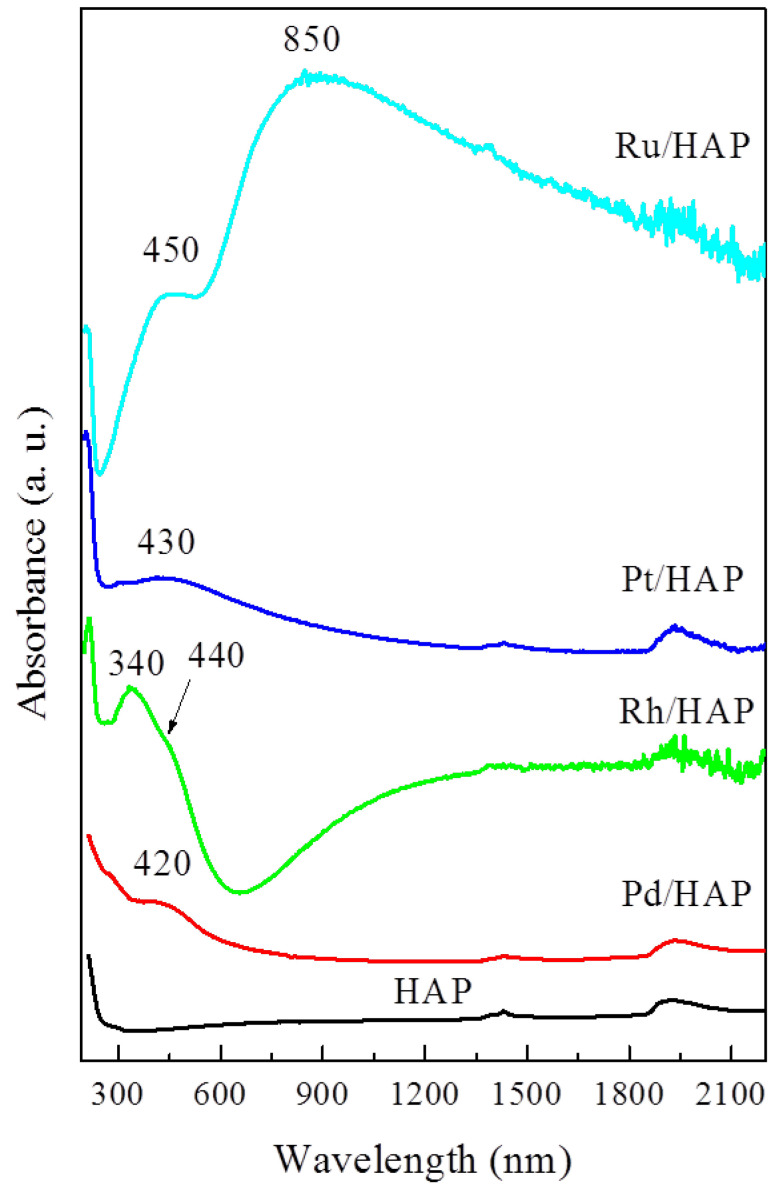
UV-Vis-NIR spectra for the noble metal catalysts.

The optical properties of the supported metallic species were investigated using UV-Vis-NIR spectroscopy. Figure 2 shows the corresponding absorption spectra. The spectrum of the HAP support exhibits a strong UV absorption peaked at 200 nm and a shoulder near 280 nm, assigned to O^2−^ → Ca^2+^ charge transfers. Irrespective of the nature of the added metal phase, these UV bands appear to intensify markedly, thereby indicating the contribution of O^2−^ → M^x+^ charge transfers. Moreover, new bands appear in the visible domain due to the d-d transitions of the cationic forms of the impregnated metals. Hence, the Pd/HAP sample exhibits a band centered at 420 nm, assigned to d-d (*ν*_1_ + *ν*_2_) transitions of Pd^2+^ ions in a tetrahedral coordination [1,7]. The spectrum of the Rh/HAP sample contains a strong absorption peaked at 340 nm accompanied by a shoulder near 440 nm. According to our previous study, the observed shape and positions indicate the main occurrence of Rh_2_O_3_ species [18]. The spectrum of the Pt/HAP sample is characterized by the presence of a much less intense visible band located at 430 nm, thus suggesting a deposition of relatively smaller amounts of the Pt cationic species. The position of this feature is very close to that observed on the spectrum of a PtO_2_ phase, as reported by Lietz et al. [25]. The deposition of Ru on the HAP support induces the occurrence of a broad absorption band extended from the visible to the NIR region and peaked at 850 nm. Xiao et al. [26] observed a similar feature, which was assigned to hydrous RuO_2_ nanoparticles (RuO_2_·xH_2_O).

**Figure 3:**
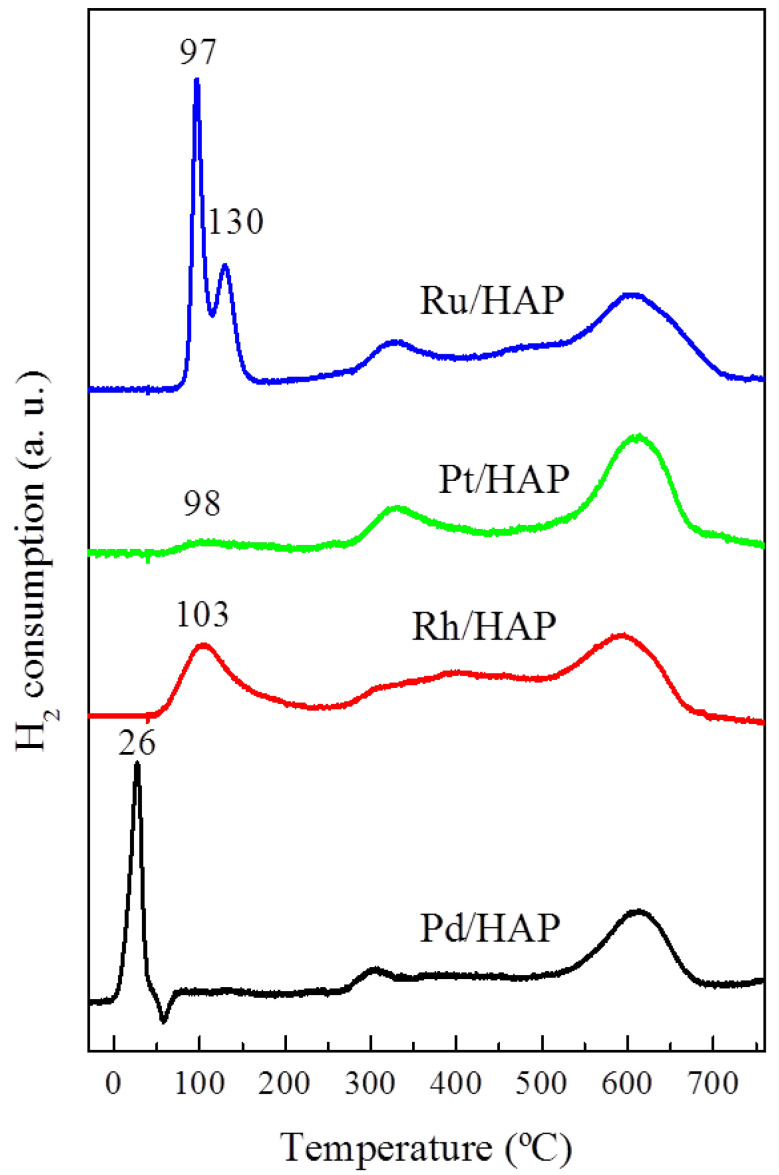
H_2_-TPR diagrams for the noble metal catalysts.

The reducibility of the prepared catalysts was investigated by H_2_-TPR. The corresponding profiles and their quantification data are included in Figure 3 and Table 1, respectively. The Pd/HAP catalyst exhibits a typical diagram of the reduction of supported PdO species [7]. The first uptake, which peaked at 26 °C, is ascribed to the PdO phase whereas the second, located at 58 °C (negative peak), is due to the decomposition of palladium hydride (Pd-H). The profile of the Rh/HAP catalyst presents a broad reduction peak centered at 103 °C. This position is comparable to that found in a previous study on Rh/Al_2_O_3_ and Rh/CeO_2_ systems [27]. In good agreement with the UV-Vis-NIR data, the amounts of consumed H_2_ suggest that the oxidized Rh species is mainly composed of the Rh_2_O_3_ phase (Table 1). The H_2_-TPR profile of the Pt/HAP catalyst is characterized by the presence of a much less intense reduction peak located at 98 °C. Moreover, the estimated H_2_/Pt molar ratio does not exceed 0.4 (Table 1). This result could be explained by the presence of additional species, probably exhibiting a strong interaction with the support that require a high reduction temperature [22]. Instead, according to the UV-Vis-NIR data, these results indicate the deposition of relatively small amounts of oxidized Pt species. The H_2_-TPR profile for the Ru/HAP sample displays two reduction peaks located at 97 and 130 °C. These features could be assigned to the reduction of two distinct ruthenium oxide species. Generally, the low temperature peak is attributed to RuO_2_ phase, whereas the peak at relatively higher temperatures can be associated with Ru species exhibiting a stronger interaction with the support [28]. The quantification of H_2_ consumption reveals that the calculated H_2_/Ru molar ratio is close to that corresponding to a stoichiometric RuO_2_ phase (Table 1). It is worth noting that the reduction temperatures of the Ru species found on our Ru/HAP sample are significantly lower than those observed on other catalyst supports [28,29]. Lanza et al. [29] attributed this effect to the occurrence of easily reducible species (RuO_2-y_(OH)*_y_*), resulting from the interaction of the RuO_2_ species with surface hydroxyl groups.

**Figure 4:**
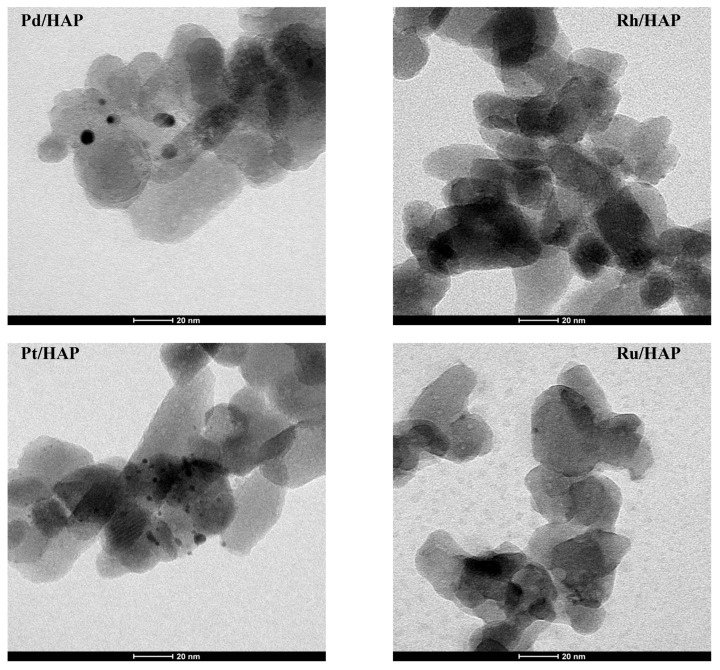
TEM micrographs of the reduced noble metal catalysts.

Figure 4 includes TEM images of the catalysts reduced at 400 °C. In all cases, quasi-spherical metallic particles can be observed. Table 2 summarizes the average particle size and the dispersion of supported samples. Interestingly, the estimated average size (d_M_) is found to depend on the nature of the active metal following this order: d_Rh_ (1 nm) < d_Pt_ (2 nm) < d_Pd_ (4.6 nm) < d_Ru_ (8.3 nm). Although the dispersion data estimated by CO chemisorptions (Table 2) differ from those given by TEM, the general tendency is found to be maintained, irrespective of the technique used.

**Figure 5:**
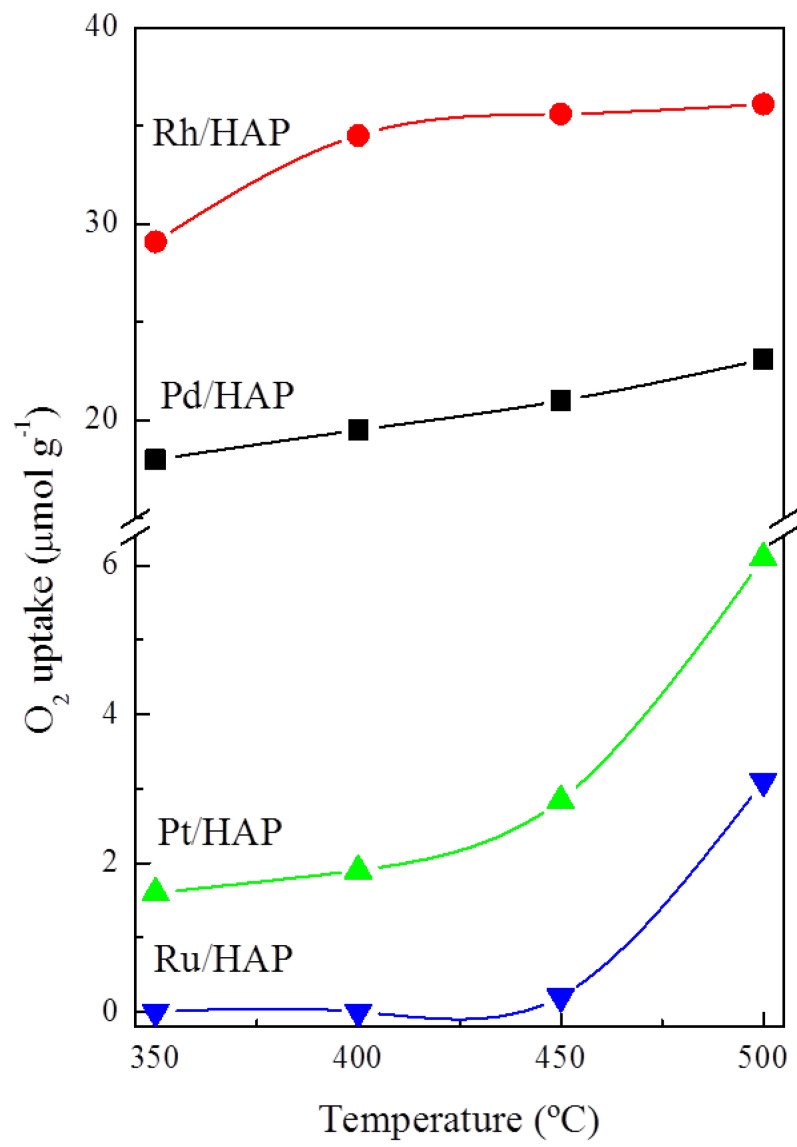
OSC studies on the noble metal catalysts.

The results corresponding to the OSC experiments, in the temperature range between 350 and 500 °C, are displayed in Figure 5. These studies provide valuable information about the ability of the reduced catalysts to be re-oxidized. It should be noted that the samples were pretreated under a reducing atmosphere (5% H_2_/Ar) at 500 °C for 1 h. As expected, over all analyzed samples the OSC activity increases with the temperature. Moreover, a comparison of the OSC values for the different catalysts reveals that they follow this general trend: Rh/HAP > Pd/HAP > Pt/HAP > Ru/HAP. Interestingly, at 500 °C, the oxygen uptakes measured on the Pd and Rh catalysts (23.1 and 36.1 μmol_O2_ g^−1^, respectively) are very similar to the theoretical values required for the formation of stoichiometric PdO and Rh_2_O_3_ phases, respectively. However, regardless of the analysis temperature, the Pt and Ru catalysts show very low OSC. Despite increasing the temperature to 500 °C, the amounts of stored oxygen do not exceed 6.1 and 3.1 μmol_O2_ g^−1^, respectively, thereby suggesting that their complete oxidation is a very slow process which probably requires higher temperatures. Instead, this low OSC activity can be related to the oxygen storage sites located at the near surface only.

### 3.2. Catalytic Activity

**Figure 6:**
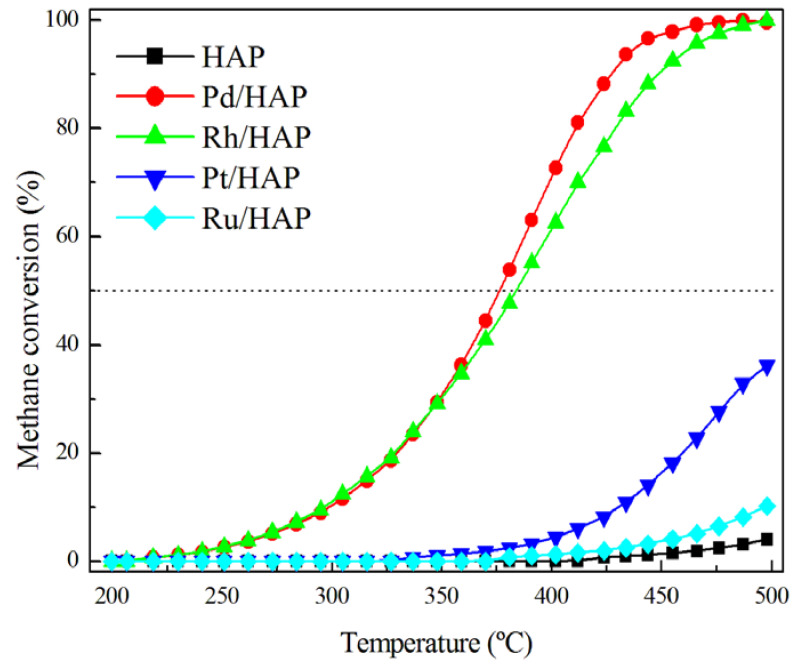
Comparison of the performance of the prepared catalysts in the methane oxidation. The reaction mixture was composed of 1% CH_4_, 20% O_2_, and 79% He.

Figure 6 displays the light-off curves of the prepared catalysts in the methane oxidation reaction. Note that carbon dioxide was the main carbonaceous product detected and only negligible amounts of carbon monoxide could be observed. As expected, the HAP bare support shows a low activity, which does not reach 4% at 500 °C. Over the Pd/HAP and Rh/HAP samples, methane conversion starts at 225 °C and increases slowly with the temperature to reach 30% at 350 °C. At higher temperatures, the Pd/HAP catalyst becomes more efficient than the Rh/HAP sample. Nevertheless, over the latter the total oxidation of methane can be reached when raising the reaction temperature to 500 °C. However, the Pt/HAP and Ru/HAP samples exhibit a poorer performance, because methane oxidation is only visible above 350 °C and the conversion levels at 500 °C do not exceed 35% and 15%, respectively. Thus, the catalyst efficiency follows this general trend: Pd/HAP > Rh/HAP > Pt/HAP > Ru/HAP. This is in good agreement with the literature data, in which palladium catalysts are generally regarded as the most promising candidates for complete methane oxidation [30,31,32,33]. For instance, a similar trend was reported by Oh et al. [30] in their study on the activity of γ-Al_2_O_3_-supported catalysts under both oxidizing and reducing conditions (Pd > Rh > Pt). On the other hand, when subjected to a second light-off experiment, the Pd/HAP and Rh/HAP catalysts show a moderate deactivation process (Figure S2). Thereafter, they maintain a stable performance.

Because there is no correlation between the performance and the metallic dispersion, it can be concluded that the dependence of methane oxidation on the metallic particle size, spread on the HAP support, is secondary. Instead, the superiority of Pd/HAP and Rh/HAP samples can be explained by their greater OSC activity. This high tendency to be re-oxidized probably generates more active metal oxides. Previous reports demonstrated that the activity of noble metals in methane oxidation proceed through the Mars–Van Krevelen mechanism, in which their ability to undergo redox processes under the reaction mixture is a determining factor [13,31,32]. Accordingly, the Ru/HAP and Pt/HAP catalysts showed a low activity, because a large fraction of the supported metal probably remains in reduced forms after being exposed to the reaction mixture, even in the presence of a high concentration of oxygen (20%).

**Figure 7:**
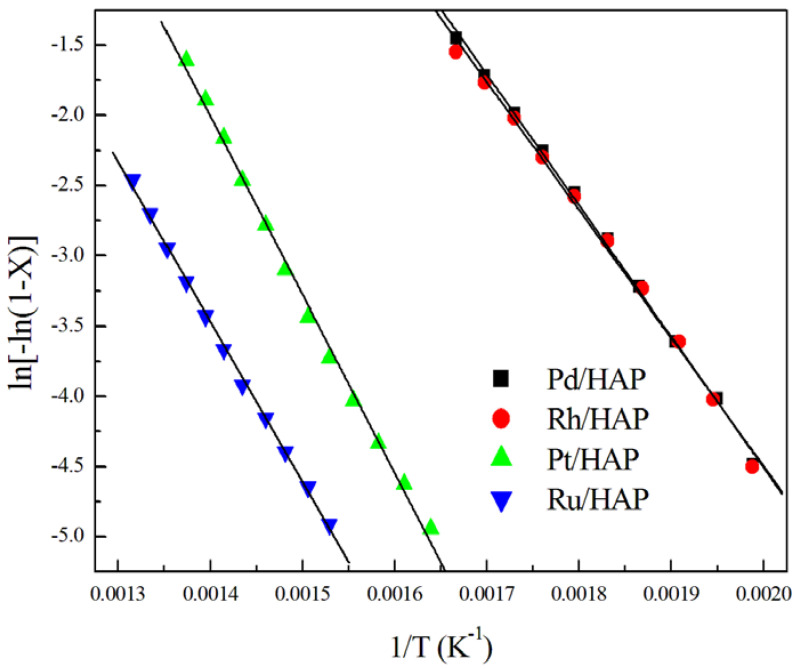
Arrhenius plots for methane oxidation over the noble metal catalysts.

The apparent activation energy (E_a_) values were estimated assuming a first order reaction. Thus, a linear correlation can be observed between ln[-ln(1-X_CH4_)] and 1/T (Figure 7). As expected, the most active catalysts (Pd/HAP and Rh/HAP) show the lowest E_a_ values (76–77 kJ mol^−1^) compared with those estimated over Pt/HAP and Ru/HAP catalysts (105 and 95 kJ mol^−1^, respectively) (Table 2). Note that our estimated values are comparable to those reported over noble metals supported on different materials [31,32,33,34,35].

**Figure 8:**
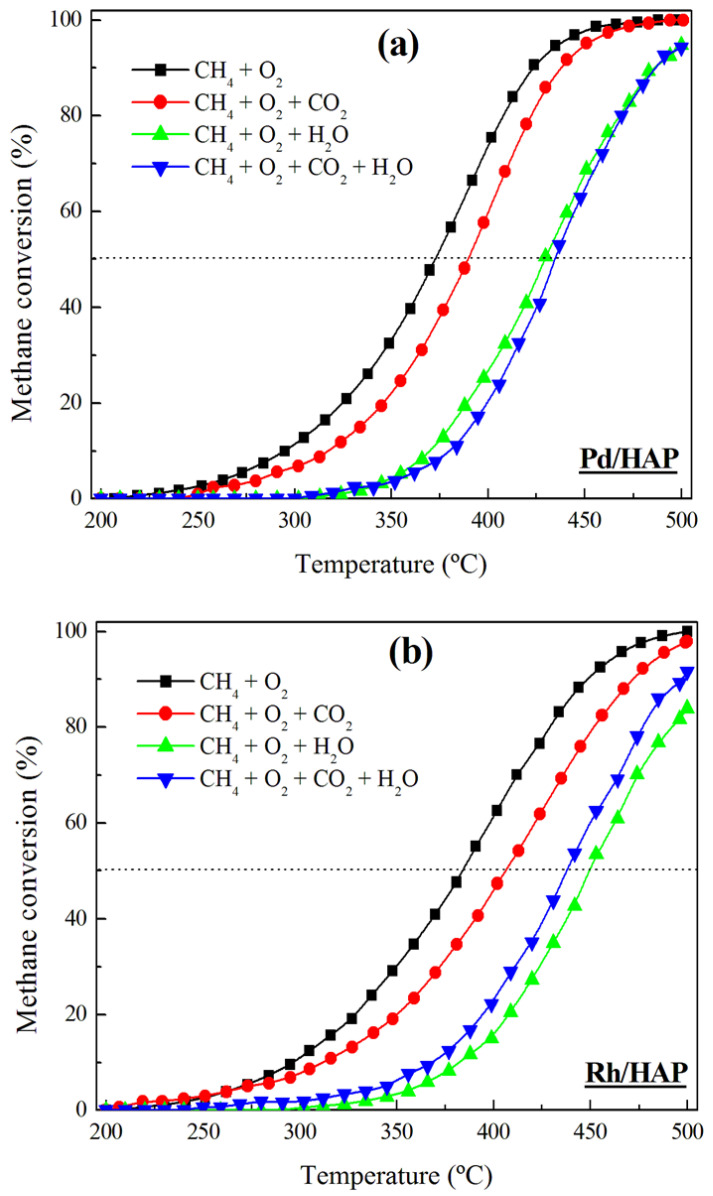
Influence of the reaction mixture composition on the performance of (**a**) Pd/HAP and (**b**) Rh/HAP catalysts in methane oxidation.

Given the promising performance of the Pd/HAP and Rh/HAP samples, additional experiments were carried out to study the effect of the addition of H_2_O and CO_2_ on their catalytic efficiency (Figure 8). Irrespective of the reaction mixture composition (CH_4_/O_2_, CH_4_/O_2_/CO_2_, CH_4_/O_2_/H_2_O, and CH_4_/O_2_/CO_2_/H_2_O), the Pd/HAP sample exhibits a higher activity than the Rh/HAP catalyst. The addition of CO_2_ causes a negative effect on the behavior of the two catalysts, which can be associated with the accumulation of carbonates on the catalyst surface [36]. However, the deactivation process is found to be more profound with the addition of water. At 400 °C, feeding water provokes a significant drop in the conversion over the Pd/HAP sample (from 75% to 25%). Similarly, over the Rh/HAP catalyst the conversion decreases from 63% to 15%. According to previous studies, the presence of water causes a severe hydroxylation of the surface’s active sites, which inhibits the re-oxidation of the noble metal catalysts [37,38,39,40,41,42]. For instance, the deactivation of Pd by water was described as a reversible process involving the formation of the inactive Pd(OH)_2_ phase (PdO + H_2_O ↔ Pd(OH)_2_) [37,38,39,41]. Interestingly, the extent of deactivation by water appears to be much lower at higher temperatures. For instance, at 500 °C, a slight decrease in the Pd/HAP activity can be observed, from 100% to 95%. In the case of the Rh/HAP catalyst, the effect of water is somewhat more appreciable because the conversion levels decrease from 100% to 84% at 500 °C. A simultaneous feeding of water and CO_2_ influences the performance of the two catalysts via different pathways. Over the Pd/HAP sample, the conversion values are slightly lower than those observed when adding water alone. By contrast, in the presence of water and CO_2_ the activity of the Rh/HAP sample is higher than that observed when adding water alone. It should be highlighted that in all cases the extent of the inhibiting effect of water and CO_2_ is significantly lower when working at high temperatures (>475 °C). Florén et al. [36] reported a similar effect in their study on the activity of a Pd/Al_2_O_3_ catalyst in a methane oxidation reaction. They associated this behavior, observed at high temperatures, with the low coverage of bicarbonates and adsorbed water, which hinder the dissociative adsorption of methane on the Pd-O site pair.

**Figure 9:**
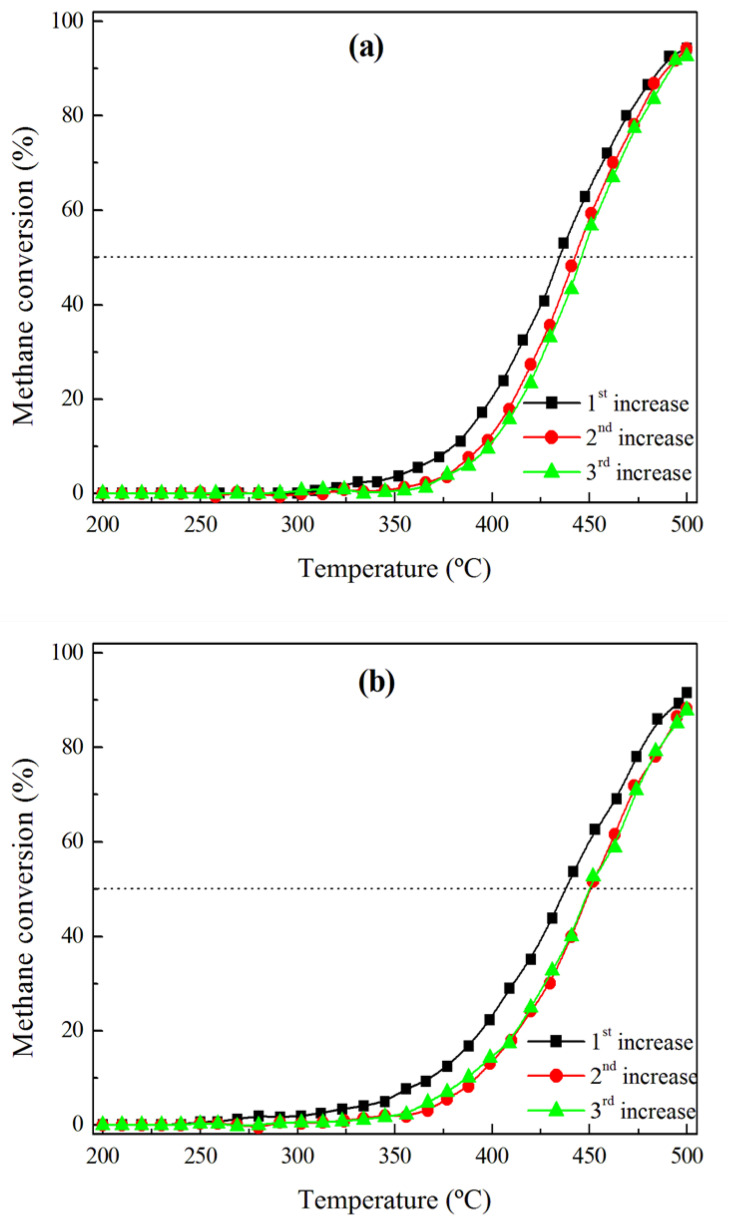
Activity of (**a**) Pd/HAP and (**b**) Rh/HAP catalysts submitted to three cycles of a realistic methane oxidation mixture: 1% CH_4_, 20% O_2_, 10% H_2_O, and 10% CO_2_.

Figure 9 shows the evolution of the activity of the Pd and Rh catalysts submitted to three consecutive cycles with the realistic reaction mixture, including water and CO_2_. With reference to the first heating ramp, the activity significantly decreases during the subsequent heating step, because T_50_ increases from 433 to 441 °C and from 436 to 449 °C over the Pd/HAP and Rh/HAP catalysts, respectively. The CO chemisorption data for the spent catalysts (Table 2) suggest that the observed deactivation can be related to a loss in the number of the exposed active sites. Thereafter, the two catalysts appear to maintain a stable performance, in which a virtually negligible difference could be observed between the second and the third heating cycles.

## 4. Conclusions

The methane oxidation reaction was investigated over various HAP supported noble metal catalysts. H_2_-TPR analysis indicated that, among all analyzed samples, the Pt catalyst exhibited the lowest reducibility. TEM and CO chemisorption studies evidenced the deposition of highly dispersed metallic Rh and Pt particles, which were significantly smaller than Pd and Ru particles. Furthermore, the OSC data revealed that although Pd and Rh catalysts could be easily re-oxidized, the re-oxidation of Pt and Ru catalysts appeared to be a slow process, resulting in small numbers of metal oxide active sites.

The catalytic results of the methane oxidation over the series of noble metals showed a better efficiency of the Pd/HAP and Rh/HAP catalysts compared with Pt/HAP and Ru/HAP samples. The superiority of the former was assigned to their high ability to undergo the redox process under the reaction mixture conditions. Feeding water and CO_2_ was found to have a negative effect, which was more pronounced in the presence of water, on the activity of Pd and Rh catalysts. However, the inhibiting effect of CO_2_ and H_2_O decreased by increasing the reaction temperature.

## Figures and Tables

**Table 1:** Characterization data of the noble metal catalysts.

Sample	BET			H_2_-TPR		
	S_BET_, m^2^ g^−1^	V_p_ ^(a)^, cm^3^ g^−1^	d_p_ ^(b)^, nm	mmol_H2_ g^−^^1^	(H_2_/M) ^(c)^	(H_2_/M) ^(d)^
HAP	55	0.40	27.8	–	–	–
Pd/HAP	53	0.35	26.5	3.8 10^−2^	0.8	1
Rh/HAP	51	0.40	29.7	6.2 10^−2^	1.3	1.5
Pt/HAP	51	0.37	28.7	1.1 10^−2^	0.4	2
Ru/HAP	48	0.36	29.1	8.7 10^−2^	1.8	2

^(a)^ Pore volume, as determined by application of the BJH method. ^(b)^ Mean pore diameter, as determined by application of the BJH method. ^(c)^ Actual H_2_/M molar ratio as determined from the integration of the reduction peaks at T < 250 °C. ^(d)^ Theoretical H_2_/M molar ratio required for the reduction of stoichiometric PdO, Rh_2_O_3_, PtO_2_, and RuO_2_.

**Table 2:** CO chemisorption, TEM, and catalytic activity data for the noble metal catalysts.

Catalyst	CO Chem	TEM		Methane Oxidation Reaction ^(b)^			
	D_Chem_, %	D_TEM_, %	d_M_, nm	T_10_	T_50_	T_100_	Ea, kJ/mol
Pd/HAP	29 (22) ^(a)^	18.6	4.6	300	375	475	77
Rh/HAP	96 (81) ^(a)^	73	1	300	390	500	76
Pt/HAP	47	41.2	2	430	–	–	105
Ru/HAP	4.7	8.2	8.3	500	–	–	95

^(a)^ Data corresponding to the catalysts submitted to three cycles of the reaction (Figure 9). ^(b)^ Data extracted from Figure 6.

## Data Availability

Data sharing is not applicable to this article.

## Supplementary Materials

The following are available online at https://www.mdpi.com/article/10.3390/ma14133612/s1, Table S1. XRD data for the noble metal catalysts; Figure S1. N_2_ physisorption isotherms for the prepared catalysts; Figure S2. Activity of (**a**) Pd/HAP and (**b**) Rh/HAP catalysts submitted to three cycles of methane oxidation reaction.

## References

[1] Boukha Z., Kacimi M., Ziyad M., Ensuque A., Bozon-Verduraz F. (2007). Comparative study of catalytic activity of Pd loaded hydroxyapatite and fluoroapatite in butan-2-ol conversion and methane oxidation. J. Mol. Catal. A Chem..

[2] Friberg I., Sadokhina N., Olsson L. (2019). The effect of Si/Al ratio of zeolite supported Pd for complete CH_4_ oxidation in the presence of water vapor and SO_2_. Appl. Catal. B Environ..

[3] Friberg I., Sadokhina N., Olsson L. (2018). Complete methane oxidation over Ba modified Pd/Al_2_O_3_: The effect of water vapor. Appl. Catal. B Environ..

[4] Gholami R., Alyani M., Smith K.J. (2015). Deactivation of Pd catalysts by water during low temperature methane oxidation relevant to natural gas vehicle converters. Catalysts.

[5] Mihai O., Smedler G., Nylén U., Olofsson M., Olsson L. (2017). The effect of water on methane oxidation over Pd/Al2O3 under lean, stoichiometric and rich conditions. Catal. Sci. Technol..

[6] Petrov A.W., Ferri D., Krumeich F., Nachtegaal M., Van Bokhoven J.A., Kröcher O. (2018). Stable complete methane oxidation over palladium based zeolite catalysts. Nat. Commun..

[7] Boukha Z., Choya A., Cortés-Reyes M., de Rivas B., Alemany L.J., González-Velasco J.R., Gutiérrez-Ortiz J.I., López-Fonseca R. (2020). Influence of the calcination temperature on the activity of hydroxyapatite-supported palladium catalyst in the methane oxidation reaction. Appl. Catal. B Environ..

[8] Petrov A.W., Ferri D., Kröcher O., Van Bokhoven J.A. (2019). Design of stable palladium-based zeolite catalysts for complete methane oxidation by postsynthesis zeolite modification. ACS Catal..

[9] Yoshida H., Nakajima T., Yazawa Y., Hattori T. (2007). Support effect on methane combustion over palladium catalysts. Appl. Catal. B Environ..

[10] Kul Ryu C., Wong Ryoo M., Soo Ryu I., Kyu Kang S. (1999). Catalytic combustion of methane over supported bimetallic Pd catalysts: Effects of Ru or Rh addition. Catal. Today.

[11] Stoian M., Rogé V., Lazar L., Maurer T., Védrine J.C., Marcu I.-C., Fechete I. (2021). Total oxidation of methane on oxide and mixed oxide ceria-containing catalysts. Catalysts.

[12] Colussi S., Trovarelli A., Cristiani C., Lietti L., Groppi G. (2012). The influence of ceria and other rare earth promoters on palladium-based methane combustion catalysts. Catal. Today.

[13] Cullis C.F., Willatt B.M. (1983). Oxidation of methane over supported precious metal catalysts. J. Catal..

[14] Boukha Z., Ayastuy J.L., Cortés-Reyes M., Alemany L.J., González-Velasco J.R., Gutiérrez-Ortiz M.A. (2019). Catalytic performance of Cu/hydroxyapatite catalysts in CO preferential oxidation in H_2_ -rich stream. Int. J. Hydrogen Energ..

[15] Boukha Z., Ayastuy J.L., Cortés-Reyes M., Alemany L.J., Gutiérrez-Ortiz M.A., González-Velasco J.R. (2018). Catalytic properties of cobalt-promoted Pd/HAP catalyst for CO-cleanup of H2-rich stream. Int. J. Hydrogen Energ..

[16] Boukha Z., Ayastuy J.L., González-Velasco J.R., Gutiérrez-Ortiz M.A. (2018). Water-gas shift reaction over a novel Cu-ZnO/HAP formulation: Enhanced catalytic performance in mobile fuel cell applications. Appl. Catal. A Gen..

[17] Boukha Z., Ayastuy J.L., González-Velasco J.R., Gutiérrez-Ortiz M.A. (2017). CO elimination processes over promoter-free hydroxyapatite supported palladium catalysts. Appl. Catal. B Environ..

[18] Boukha Z., Gil-Calvo M., de Rivas B., González-Velasco J.R., Gutiérrez-Ortiz J.I., López-Fonseca R. (2018). Behaviour of Rh supported on hydroxyapatite catalysts in partial oxidation and steam reforming of methane: On the role of the speciation of the Rh particles. Appl. Catal. A Gen..

[19] Boukha Z., González-Prior J., de Rivas B., González-Velasco J.R., López-Fonseca R., Gutiérrez-Ortiz J.I. (2018). Pd supported catalyst for gas-phase 1,2-dichloroethane abatement: Efficiency and high selectivity towards oxygenated products. J. Ind. Eng. Chem..

[20] Boukha Z., González-Prior J., Rivas B., González-Velasco J.R., López-Fonseca R., Gutiérrez-Ortiz J.I. (2016). Synthesis, characterisation and behaviour of Co/hydroxyapatite catalysts in the oxidation of 1,2-dichloroethane. Appl. Catal. B Environ..

[21] Boukha Z., González-Velasco J.R., Gutiérrez-Ortiz M.A. (2021). Exceptional performance of gold supported on fluoridated hydroxyapatite catalysts in CO-cleanup of H_2_-rich stream: High activity and resistance under PEMFC operation conditions. Appl. Catal. B Environ..

[22] Boukha Z., González-Velasco J.R., Gutiérrez-Ortiz M.A. (2020). Platinum supported on lanthana-modified hydroxyapatite samples for realistic WGS conditions: On the nature of the active species, kinetic aspects and the resistance to shut-down/start-up cycles. Appl. Catal. B Environ..

[23] Boukha Z., Kacimi M., Pereira M.F.R., Faria J.L., Figueiredo J.L., Ziyad M. (2007). Methane dry reforming on Ni loaded hydroxyapatite and fluoroapatite. Appl. Catal. A-Gen..

[24] Boukha Z., Yeste M.P., Cauqui M.Á., González-Velasco J.R. (2019). Influence of Ca/P ratio on the catalytic performance of Ni/hydroxyapatite samples in dry reforming of methane. Appl. Catal. A-Gen..

[25] Lietz G., Lieske H., Spindler H., Hanke W., Völter J. (1983). Reactions of platinum in oxygen- and hydrogen-treated Pt γ-Al_2_O_3_ catalysts. II. Ultraviolet-visible studies, sintering of platinum, and soluble platinum. J. Catal..

[26] Xiao Z., Jiang X., Li B., Liu X., Huang X., Zhang Y., Ren Q., Luo J., Qin Z., Hu J. (2015). Hydrous RuO_2_ nanoparticles as an efficient NIR-light induced photothermal agent for ablation of cancer cells in vitro and in vivo. Nanoscale.

[27] Varga E., Baán K., Samu G.F., Erdőhelyi A., Oszkó A., Kónya Z., Kiss J. (2016). The effect of Rh on the interaction of Co with Al_2_O_3_ and CeO_2_ supports. Catal. Lett..

[28] Jones D.R., Iqbal S., Kondrat S.A., Lari G.M., Miedziak P.J., Morgan D.J., Parker S.F., Hutchings G.J. (2016). An investigation of the effect of carbon support on ruthenium/carbon catalysts for lactic acid and butanone hydrogenation. Phys. Chem. Chem. Phys..

[29] Lanza R., Järås S.G., Canu P. (2007). Partial oxidation of methane over supported ruthenium catalysts. Appl. Catal. A-Gen..

[30] Oh S.H., Mitchell P.J., Siewert R.M. (1991). Methane oxidation over alumina-supported noble metal catalysts with and without cerium additives. J. Catal..

[31] Hurtado P., Ordóñez S., Sastre H., Díez F.V. (2004). Development of a kinetic model for the oxidation of methane over Pd/Al_2_O_3_ at dry and wet conditions. Appl. Catal. B Environ..

[32] Todorova S., Naydenov A., Kolev H., Ivanov G., Ganguly A., Mondal S., Saha S., Ganguli A.K. (2018). Reaction kinetics and mechanism of complete methane oxidation on Pd/Mn_2_O_3_ catalyst. React. Kinet. Mech. Catal..

[33] Wonoputri V., Effendy M., Wibisono Budhi Y., Bindar Y. (2013). Determination of kinetic parameters for methane oxidation over Pt/γ-Al_2_O_3_ in a fixed-bed reactor. J. Eng. Technol. Sci..

[34] Garetto T.F., Apesteguía C.R. (2000). Oxidative catalytic removal of hydrocarbons over Pt/Al_2_O_3_ catalysts. Catal. Today.

[35] Okal J., Zawadzki M. (2013). Catalytic combustion of methane over ruthenium supported on zinc aluminate spinel. Appl. Catal. A Gen..

[36] Florén C.-R., Van Den Bossche M., Creaser D., Grönbeck H., Carlsson P., Korpi H., Skoglundh M. (2018). Modelling complete methane oxidation over palladium oxide in a porous catalyst using first-principles surface kinetics. Catal. Sci. Technol..

[37] Gélin P., Primet M. (2002). Complete oxidation of methane at low temperature over noble metal based catalysts: A review. Appl. Catal. B Environ..

[38] Kikuchi R., Maeda S., Sasaki K., Wennerström S., Eguchi K. (2002). Low-temperature methane oxidation over oxide-supported Pd catalysts: Inhibitory effect of water vapor. Appl. Catal. A Gen..

[39] Eriksson S., Boutonnet M., Järås S. (2006). Catalytic combustion of methane in steam and carbon dioxide-diluted reaction mixtures. Appl. Catal. A Gen..

[40] Mao Q., van Duin A.C.T., Luo K.H. (2017). Investigation of methane oxidation by palladium-based catalyst via ReaxFF molecular dynamics simulation. Proc. Combust. Inst..

[41] Li X., Wang X., Roy K., Van Bokhoven J.A., Artiglia L. (2020). Role of Water on the structure of palladium for complete oxidation of methane. ACS Catal..

[42] Zhang Y., Glarborg P., Johansen K., Andersson M.P., Torp T.K., Jensen A.D., Christensen J.M. (2020). A rhodium-based methane oxidation catalyst with high tolerance to H_2_O and SO_2_. ACS Catal..

