# Supplementary material for: Comparative Study of the Efficiency of Different Noble Metals Supported on Hydroxyapatite in the Catalytic Lean Methane Oxidation under Realistic Conditions
Source: Materials (Basel). 2021 Jun 28;14(13):3612. doi: 10.3390/ma14133612 (PMC8269712; doi:10.3390/ma14133612)
Supplement: Supplementary file 1 [file materials-14-03612-s001.zip › materials-1254089-supplementary.pdf]

materials

MDPI

Comparative Study of the Efficiency of Different Noble Metals Supported on Hydroxyapatite in the Catalytic Lean Methane Oxidation under Realistic Conditions

Zouhair Boukha *, Beatriz de Rivas, Juan R. González-Velasco, José I. Gutiérrez-Ortiz and Rubén López-Fonseca

Chemical Technologies for Environmental Sustainability Group, Chemical Engineering Department, Faculty of Science and Technology, University of The Basque Country UPV/EHU, E-48940 Leioa, Bizkaia, Spain; beatriz.derivas@ehu.eus (B.d.R.); juanra.gonzalezvelasco@ehu.eus (J.R.G.-V.); joseignacio.gutierrez@ehu.eus (J.I.G.-O.); ruben.lopez@ehu.eus (R.L.-F.)

* Correspondence: zouhair.boukha@ehu.eus

Table S1. XRD data for the noble metal catalysts.

Sample	XRD[(a)]		
	a, Å	c, Å	V, Å^3
HAP	9.4148	6.8791	528.1
Pd/HAP	9.4124	6.8801	527.9
Rh/HAP	9.4087	6.8773	527.2
Pt/HAP	9.4108	6.8785	527.6
Ru/HAP	9.4098	6.8792	527.5

(a) HAP lattice parameters

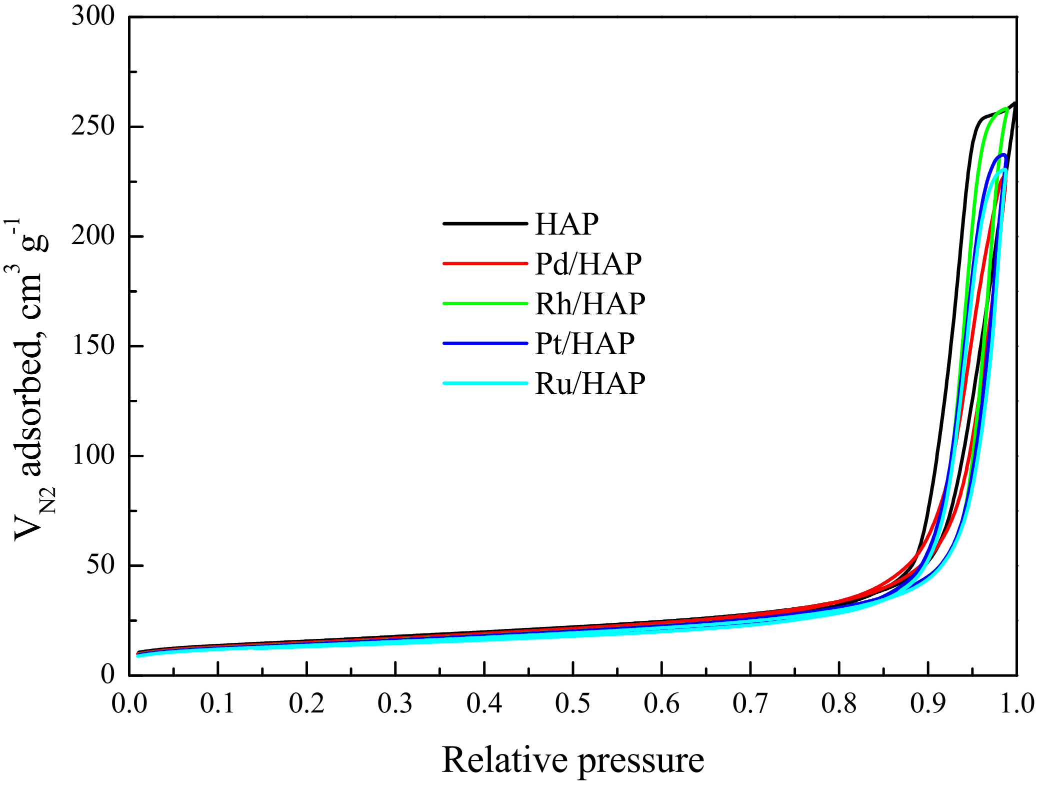

Figure S1. N_2 physisorption isotherms for the prepared catalysts.

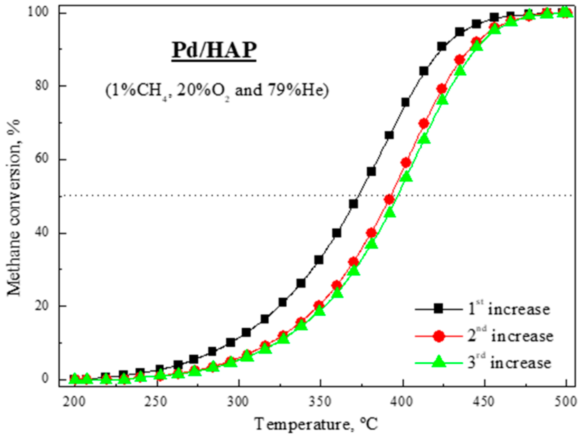

(**a**)

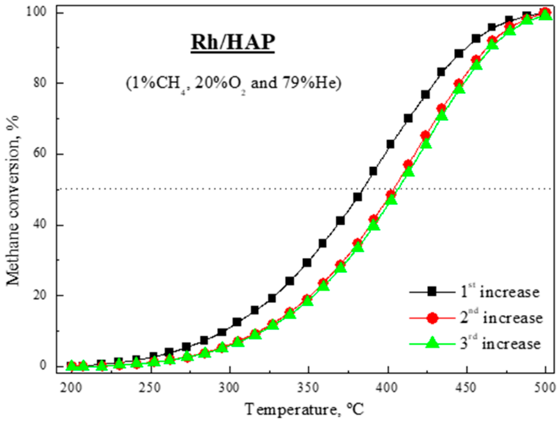

(**b**)

Figure S2. Activity of (**a**) Pd/HAP and (**b**) Rh/HAP catalysts submitted to three cycles of methane oxidation reaction.
